# King Edward's Hospital Fund: Annual Meeting and Report

**Published:** 1922-05

**Authors:** 


### KING EDWARD'S HOSPITAL FUND FOR LONDON.

### THE ANNUAL MEETING AND REPORT.

THE Annual Meeting of the President and General Council of King Edward's Hospital Fund for London, to receive the accounts and report for the year 1921, was held at the House of Lords on March 28, the Earl of Donoughmore being in the chair.

Lord Somerleyton (hon. secretary) said that since the Council last met on March 8, the Organising Committee, of which Sir Alan Anderson is chairman, had been constituted and included the Revenue Committee of the King's Fund and representatives of the Hospital Sunday and Hospital Saturday Funds, the Joint Council of the Order of St. John and the British Red Cross Society, the League of Mercy, and the London Regional Committee of the British Hospitals Association. The Trustees and Treasurers of the Appeal Fund were the Lord Mayor of London, the Governor of the Bank of England, the Chairman of the London Regional Committee of the British Hospitals Association, and the Treasurer of the King's Fund. The Organising Committee had appointed an Executive Committee under the chairmanship of Sir George Lawson-Johnston, and had appointed as Director-General of the Appeal Campaign Mr. E. S. Shrapnell-Smith, and Captain E. J. C. Chapman as his deputy. The appeal would be launched after Easter, and would reach its climax by June. Each hospital on the list of the King's Fund as Voluntary Hospitals Committee for London had been invited to co-operate, and from all quarters the Organising Committee had received the most cordial assurances of support.

The Combined Appeal aimed at raising at least £500,000, needed to pay off the floating debt of London hospitals. Its second object was to secure for hospitals the new subscribers and the additional permanent income to meet the increased cost of working, including moderate and continuous contributions from all classes as recommmended by Lord Cave's Committee. It was not being made by the King's Fund alone, but mobilised the influence of the principal organisations working on behalf of the hospitals, and above all, of the hospitals themselves.

The report of the Management Committee was adopted on the subject of expenses for 1921, which, in spite of the increased work, still averaged less than 6¼d. in every pound received.

Lord Revelstoke (hon. treasurer), in presenting the Account of Receipts and Expenditure and the Balance Sheet for the year ended December 31, 1921, said that the total general receipts amounted to £252,659 6s. 2d. Owing to the receipt of several exceptional legacies, these figures showed a considerable increase as compared with those of 1920. During the past year they had distributed £220,000, an amount exceeding by £20,000 that distributed in 1920; and they had added £25,486 14s. 4d. to the reserves. In 1920 the reserves were depleted by an emergency distribution of £250,000, which was regarded as an entirely exceptional effort. It was evident that, if the King's Fund were to be instrumental in ensuring the financial well-being of the hospitals, it must maintain and increase its own permanent income. Lord Mount Stephen had had this object in view in his munificent gifts to capital during his lifetime, lately supplemented by the bequest of the residue of his estate. It was an object of paramount importance to them at a moment when they had already sacrificed a considerable amount of permanent income, and when they had to regret the loss of their largest annual subscriber, the late Mr. Walter Morrison, who for a long period of years had contributed a yearly sum of £5,000. It was satisfactory to be able to report the announcement of another important bequest. The late Sir Thomas Sutherland had appointed the King's Fund as residuary legatee to his estate. Even after the payment of the Death Duties, for which the Fund would be responsible, this bequest would form the nucleus of a valuable addition to their income.

The King's Fund now proposed to contribute to the solution of the problem entailed by the present crisis, not by making another distribution at the expense of its future income, but by the organisation, at the request of the hospitals and with the aid of the other central agencies, of a great combined public appeal. The Prince of Wales, as President of the King's Fund, had authorised the use of his name in connection with this.

Lord Cave said he thought that the report of his Committee had been very kindly received by the Government, because in many respects they acted upon their recommendations, although they did not go the whole way. As regards the Death Duties, he suggested to the Members of the House of Commons on the Council, that it was open to them to see whether the relief from Death Duties which his Committee recommended, could not, at all events in some measure, be obtained. There was also the reduction in the grant recommended for the relief of the hospitals from their present difficultes, due, he believed, entirely to the late war. There, again, the Government were not able to go the whole way, and therefore the duty had fallen upon the King's Fund to make a combined public appeal. He believed it to be necessary, not only that a considerable sum should be raised to meet the immediate necessities of the case, but that provision should be made that the like necessity should not soon recur.

The report for 1921, presented by Lord Stuart of Wortley (Chairman of the Management Committee), shows that in 1921, even more than in 1920, the history of the Fund was bound up with the history of the crisis of the hospital finances and the suggested remedies. The crisis was due to the increase in the cost of working, which had far outstripped the increase in income. It first became acute in April, 1920, and in July of that year the King's Fund made an emergency distribution of £250,000 out of its reserves, while in December, 1920, the National

Relief Fund distributed £200,000 to London hospitals in relief of war deficits. But these special distributions only just equalised the income and expenditure of the London hospitals for the year 1920, leaving unsolved the problems of the accumulated deficits of the past and the annual deficits of the future.

In June, 1921, the report of Lord Cave's Committee of Inquiry was published, and the King's Fund was actively engaged in two functions, that of temporary assistance and the continued search for permanent remedies. In July, a Mansion House Conference of London Hospitals was held to consider the suggestion by Lord Cave's Committee that the permanent solution might be found in some form of mass contribution from the hospital-using classes; and a standing conference of the King's Fund and the other Central Agencies was set up to work out a scheme. In October the Fund presented a report on the financial position of the hospitals to the Voluntary Hospitals Commission, who thereupon made an emergency distribution of £77,900, strictly limited to 24 hospitals that were in immediate danger of having to close beds. The urgency of the position was immediately reported by the King's Fund to the Conference of Central Agencies, with the result that in November the King's Fund was asked by the London Regional Committee of the British Hospitals Association, on which more than 100 hospitals in London are represented, to draw up two schemes, one for a combined appeal for fresh money to meet present liabilities and qualify for temporary Government assistance on the pound-for-pound basis, and the other for the collection of regular contributions from the hospital-using classes on the general lines of one of the alternative methods recommended by Lord Cave's Committee. When the year ended the two schemes drawn up in response to this request had been circulated by the London Regional Committee to its constituent hospitals, but it was not until January, 1922, that they were received back with the definite invitation to the King's Fund to go forward with both.

The total receipts for the year were £270,666 12s. 6d. Of this £18,007 6s. 4d. was for capital and £252,659 6s. 2d. general receipts. The amount distributed was £220,000, being an increase of £20,000 on the ordinary distribution of 1920, the total grants to hospitals in 1921 being £210,000, as against £190,000 in 1920.

In view of the financial crisis the Distribution Committee notified the hospitals in October that only in exceptional cases could they recommend grants for extension or improvement, or consider schemes not already notified to the King's Fund when evidence on this subject was given to Lord Cave's Committee in April. As a result of this policy the amount available for maintenance was increased not only by the £20,000 increase in the total distributed, but also by a decrease of £24,725 in the grants to capital expenditure, or £44,725 in all. In distributing the increased amount available for maintenance, the Distribution Committee took into account the efforts made by the hospitals to increase income or reduce expenditure since the emergency distribution in July, 1920, and the issue of Lord Cave's report in June, 1921.

The grants to consumption sanatoria in 1921 amounted to £6,070 as against £6,420 in 1920, and the grant to convalescent homes to £3,930 in 1921, as against £3,580 in 1920. The total sum distributed amongst hospitals, convalescent homes, and consumption sanatoria during the last 10 years was £2,340,938. Since the foundation of the Fund a total net amount of £3,816,854 8s. 5d. has been distributed. The average annual distribution during the whole period of 25 years has been £152,674 3s. 6d.

The King, who is Patron of the Fund, has been graciously pleased to continue his annual subscription of £1,000. The Queen, Queen Alexandra, the Prince of Wales, the Duke of York, Princess Mary, and other members of the Royal Family have also been pleased to subscribe generously. The League of Mercy contributed £15,000 during 1920, and the total received by the Fund from the League since its foundation in 1899 is now £324,000, apart from the sums awarded by the League to institutions outside the area of the Fund's distribution. The Council congratulates the League on the continued success of its efforts in the systematic collection of moderate and continuous contributions, which is one of the methods specially recommended by the Cave Committee. Legacies received during the year amounted to £79,895 5s. 9d.

The Report also deals with the reorganisation which has taken place in order to meet the new duties undertaken by the King's Fund as the Voluntary Hospitals Committee for London, which have meant a great increase in its work and consequently in its expenditure. The Committees of the Fund were reorganised in order to secure the efficient performance of their functions, and the new scheme of administration came into operation on January 1, 1922.

During the year the amount spent on the administration of the Fund was £7,208 13s. 6d., or £2 11s. 8d. per £100 of the total received. The average yearly outlay in this respect for the whole period since the inauguration of the Fund had been £3,225—less than 3¼d. in every £ received.

The Council records with pleasure the stimulus which the publication of Lord Cave's report has given to the movement for co-operation between the Hospitals and the various Central Agencies, and expresses a hope that the combined appeal to meet the present emergency will bring permanent benefit, not only to the common cause, but also to each separate agency.

---

The grants made by the Voluntary Hospitals Commission now amount to over £127,000. Recent grants include those to the Bristol Royal Infirmary (for re-opening closed beds), £3,350; Sheffield Royal Infirmary, £10,000; Sheffield Royal Hospital, £7,650; Royal Albert Hospital, Devonport, £1,000; Middlesex Hospital, £5,000; West London Hospital, £3,500; and the Prince of Wales' Hospital, Tottenham, £1,800. Hospitals which had a deficit on maintenance account for 1921 should take steps to complete their accounts without delay, so that they may be in a position to apply for grants through their local Hospital Committees.

